# Biomechanical factors and physical examination findings in osteoarthritis of the knee: associations with tissue abnormalities assessed by conventional radiography and high-resolution 3.0 Tesla magnetic resonance imaging

**DOI:** 10.1186/ar4050

**Published:** 2012-10-05

**Authors:** Jesper Knoop, Joost Dekker, Jan-Paul Klein, Marike van der Leeden, Martin van der Esch, Dick Reiding, Ramon E Voorneman, Martijn Gerritsen, Leo D Roorda, Martijn PM Steultjens, Willem F Lems

**Affiliations:** 1 Amsterdam Rehabilitation Research Center - Reade, Dr. Jan van Breemenstraat 2, 1056 AB Amsterdam, the Netherlands; 2 Department of Rehabilitation Medicine/EMGO, VU University Medical Center, De Boelelaan 1118, 1081 HZ Amsterdam, the Netherlands; 3 Department of Psychiatry, VU University Medical Center, De Boelelaan 1118, 1081 HZ Amsterdam, the Netherlands; 4 Department of Radiology, VU University Medical Center, De Boelelaan 1118, 1081 HZ Amsterdam, the Netherlands; 5 Jan van Breemen Research Institute - Reade, Dr. Jan van Breemenstraat 2, 1056 AB Amsterdam, the Netherlands; 6 School of Health and Life Sciences, Institute for Applied Health Research, Glasgow Caledonian University, Cowcaddens Road, Glasgow G4 0BA, UK; 7 Department of Rheumatology, VU University Medical Center, VU University Medical Center, De Boelelaan 1117, 1081 HV Amsterdam, the Netherlands

## Abstract

**Introduction:**

We aimed to explore the associations between knee osteoarthritis (OA)-related tissue abnormalities assessed by conventional radiography (CR) and by high-resolution 3.0 Tesla magnetic resonance imaging (MRI), as well as biomechanical factors and findings from physical examination in patients with knee OA.

**Methods:**

This was an explorative cross-sectional study of 105 patients with knee OA. Index knees were imaged using CR and MRI. Multiple features from CR and MRI (cartilage, osteophytes, bone marrow lesions, effusion and synovitis) were related to biomechanical factors (quadriceps and hamstrings muscle strength, proprioceptive accuracy and varus-valgus laxity) and physical examination findings (bony tenderness, crepitus, bony enlargement and palpable warmth), using multivariable regression analyses.

**Results:**

Quadriceps weakness was associated with cartilage integrity, effusion, synovitis (all detected by MRI) and CR-detected joint space narrowing. Knee joint laxity was associated with MRI-detected cartilage integrity, CR-detected joint space narrowing and osteophyte formation. Multiple tissue abnormalities including cartilage integrity, osteophytes and effusion, but only those detected by MRI, were found to be associated with physical examination findings such as crepitus.

**Conclusion:**

We observed clinically relevant findings, including a significant association between quadriceps weakness and both effusion and synovitis, detected by MRI. Inflammation was detected in over one-third of the participants, emphasizing the inflammatory component of OA and a possible important role for anti-inflammatory therapies in knee OA. In general, OA-related tissue abnormalities of the knee, even those detected by MRI, were found to be discordant with biomechanical and physical examination features.

## Introduction

Osteoarthritis (OA) of the knee involves many tissues of the knee joint, not only addressing cartilage but also including abnormalities in subchondral bone and the synovial membrane [1,2]. Most people with knee OA suffer from pain, stiffness and limitations in daily activities [2]. Physical examination may reveal clinical signs such as joint crepitus, swelling, deformities or increased warmth of the joint [2]. Additionally, biomechanical factors such as lower limb muscle strength, proprioceptive accuracy of the knee joint and varus-valgus knee joint laxity, which are considered essential factors for knee stabilization [3-5], have frequently been found to be impaired in knee OA patients [6-8]. Besides being clinically important consequences of OA, biomechanical factors may also play a role in the onset of tissue abnormalities [9-12]. Presumably, biomechanical factors in the knee joint and tissue abnormalities interact with each other during the disease process of OA.

Conventional radiography (CR) is the primary modality for disease diagnosis and classification in clinical practice [13]. CR-based joint space width (JSW), an indirect measure for cartilage loss, is the most important outcome measure in pharmacological studies [14]. In contrast to radiography, magnetic resonance imaging (MRI) is able to visualize cartilage and to detect bone marrow lesions (BML) and inflammation (for example, effusion and synovitis) [14]. MRI is therefore currently the best modality available for imaging OA-related tissue abnormalities [14] and may be able to unravel mechanisms underlying biomechanical impairments.

Only a few studies have been performed on the association between tissue abnormalities and biomechanical factors. While studies using CR provided mixed results [15-19], studies using MRI clearly demonstrated an association between (medial tibiofemoral and patellafemoral compartmental) cartilage thickness and quadriceps strength [18,20,21]. A small number of studies (using CR or MRI) provided mixed results on the relationship between tissue abnormalities (namely, cartilage thickness [8,22,23] and osteophyte formation [22,24]) and knee joint laxity, while proprioceptive accuracy and hamstrings muscles have never been studied in relation to tissue abnormalities.

Most studies concerning OA-related tissue abnormalities focused on the association with patient-reported pain or activity limitations, generally providing evidence for discordance - particularly in studies using CR [13,25-32]. OA-related tissue abnormalities are possibly more closely linked to findings from physical examination, rather than to self-reported outcomes. Recently, a population-based study using MRI showed that multiple tissue abnormalities were related to the presence of crepitus [33]. As far as we know, tissue abnormalities have not so far been related to findings from physical examination in a knee OA population.

In conclusion, there is limited knowledge on the association between OA-related tissue abnormalities, biomechanical factors and physical examination findings in knee OA. The first aim of the present study was therefore to explore associations of CR-detected and MRI-detected tissue abnormalities and biomechanical factors (quadriceps and hamstrings muscle strength, proprioceptive accuracy and knee joint laxity) in patients with knee OA. The second aim was to explore associations of CR-detected and MRI-detected tissue abnormalities and physical examination findings (bony tenderness, crepitus, bony enlargement and palpable warmth) in patients with knee OA.

## Materials and methods

### Subjects

For the present study, participants were recruited from a randomized controlled trial on the effectiveness of a knee stabilization exercise program [34]. Inclusion criteria were clinical knee OA diagnosis according to the American College of Rheumatology criteria (that is, presence of knee pain and at least three of the following six items: age >50 years, morning stiffness <30 minutes, crepitus, bony tenderness, bony enlargement and no palpable warmth) [35], age between 40 and 75 years, instability of the knee and written informed consent. Total knee arthroplasty, rheumatoid arthritis or any other form of arthritis (that is, crystal arthropathy, septic arthritis, spondylarthropathy), comorbidities affecting daily functioning, severe knee pain (numeric rating scale >8) and/or contraindication for MRI (for example, pacemaker, claustrophobia) were exclusion criteria. Patients were subsequently examined by radiologists, rheumatologists, and physiatrists. The measurement protocol contained assessment of demographic, biomechanical and clinical factors related to OA, as well as CR and MRI, all assessed prior to the start of the trial. All participants provided written informed consent. The study was approved by the Reade/Slotervaart Institutional Review Board.

### Index knee

For knee-specific variables we used data from the index knee. For unilateral knee OA patients, the index knee was the knee that was diagnosed with clinical OA. For bilateral knee OA patients, the index knee was the knee that most severely affected daily activities on patient self-report.

### Biomechanical factors

Measurements of lower limb muscle strength, proprioceptive accuracy and varus-valgus laxity have been extensively described in previous publications [5,36,37]. In summary, muscle strength was measured isokinetically (60°/second) for both knee extension (quadriceps) and flexion (hamstrings) strength. Strength outcomes (in Nm) were adjusted for bodyweight [36]. For proprioceptive accuracy (knee joint motion sense), a threshold detection task was used - which assessed the amount of degrees after motion detection, with motion velocity of 0.3°/second [36]. Varus-valgus knee joint laxity was measured as the total range of knee motion (in degrees) in the frontal plane. In a sitting position, the thigh and lower leg were fixed at five places to prevent for medial or lateral movement of the thigh and lower leg and for hip rotation. In a fixed knee flexion of 20°, a load of 1.12 kg (7.7 Nm) was applied to the lower leg both medially and laterally, resulting in varus or valgus movement across the transverse axis of the knee joint [37].

### Physical examination findings

The following features from the American College of Rheumatology criteria for clinical knee OA diagnosis were assessed by the physiatrist on physical examination of the knee joint: bony tenderness (that is, pain by palpation at the joint line), crepitus (that is, crackling or grinding sound in the joint during weight bearing), bony enlargement at joint line and palpable warmth of the knee joint [35]. Findings were scored as yes (present) or no (absent).

### Radiography

Conventional radiographs of tibiofemoral joints were made by a weight-bearing posterioanterior view, semi-flexed (7 to 10°) according to Buckland-Wright and colleagues [38]. Radiographs of patellofemoral joints were made by a single standing mediolateral view in 30° flexion, and a skyline (inferior superior) view in 30° flexion [39]. Two independent observers (DR, MvdE) graded radiographs, unaware of the patient's clinical characteristics. One observer (DR) was a bone and joint radiologist, and the second observer (MvdE) was an epidemiologist trained by two musculoskeletal radiologists. The JSW and osteophyte formation were scored on a scale of 0 to 3, for medial tibiofemoral (MTF), lateral tibiofemoral (LTF) and patellafemoral (PF) compartments separately, according to the Osteoarthritis Research Society International (OARSI) atlas [40]. Severity of structural damage in the knee, according to Kellgren-Lawrence [41], was also scored. The intraclass correlation coefficient for interrater reliability in 64 knees was 0.87 (*P *<0.001) for JSW, 0.60 (*P *<0.001) for osteophytes and 0.89 (*P *<0.001) for the Kellgren-Lawrence score.

### Magnetic resonance imaging

MRI scans of one knee (the index knee) were performed by a 3.0 Tesla whole-body magnetic resonance scanner (General Electric Medical Systems, Milwaukee, WI, USA) using a phased array knee coil. The MRI examination included five scans. All scans were made with a field of view of 180 mm. The first sequence was a sagittal proton density-weighted turbo spin-echo with fat suppression (slice thickness 3 mm; interslice gap 0.3 mm; repetition time 3,480 milliseconds; echo time 42 milliseconds; turbo factor 8; matrix 384×256). The second sequence was a sagittal T1-weighted turbo spin-echo (slice thickness 3 mm; interslice gap 0.3 mm; repetition time 760 milliseconds; echo time 14 milliseconds; turbofactor 2; matrix 384×256). The third sequence was a coronal T2-weighted turbo spin-echo with fat suppression (slice thickness 3mm; interslice gap 0.3 mm; repetition time 5,800 milliseconds; echo time 85 milliseconds; turbo factor 15; matrix 384×256). The fourth sequence was a sagittal combined multi-echo gradient echo (thickness 3.5 mm; interslice gap 0.3 mm; repetition time 973 milliseconds; excitation angle 20°; matrix 352×224). The last sequence was a coronal combined multi-echo gradient echo (thickness 3.0 mm; interslice gap 0.5 mm; repetition time 854 milliseconds; excitation angle 20°; matrix 352×224).

MRI scans were assessed according to the Boston-Leeds Osteoarthritis Knee Score system [42], a semi-quantitative whole-joint scoring method. A radiologist (J-PK) with 27 years of musculoskeletal expertise, blinded to the patient's clinical characteristics and radiographic assessment, assessed all MRI scans. Cartilage integrity, osteophyte formation and BML were scored per region, with scores ranging from 0 (no abnormality) to 3 (severe abnormality). For effusion, one knee-specific score was used, ranging from 0 (physiological amount of effusion) to 3 (large effusion). Presence of synovitis (yes/no) was assessed in five regions separately. Specific details on MRI assessment are presented in Table 1. The intraclass correlation coefficient for intrarater reliability in 15 knees was found to be 0.83 (*P *<0.001) for cartilage thickness, 0.86 (*P *<0.001) for osteophytes, 0.91 (*P *<0.001) for BML, and 0.97 (*P *<0.001) for effusion. Cohen's kappa for synovitis was 0.73 (*P *= 0.003).

**Table 1:** Magnetic resonance imaging assessment, according to Boston-Leeds Osteoarthritis Knee Score [42]

		Cartilage integrity	Osteophytes	Bone marrow lesions	Effusion	Synovitis
Assessment		Size of any cartilage thickness loss (both partial and full thickness loss) as percentage of cartilage surface area of each region	Size of protuberance of osteophyte	Size of BML (including volume of any associated cysts) as percentage of bone volume of each region	Size of effusion within synovial space	Presence of synovitis
Region	Number	8	12	9	1 (one score for whole knee)	5
	MTF	Medial weight-bearing femur	Medial weight-bearing femur	Medial weight-bearing femur	-	**-**
		Medial region tibia	Medial posterior region femur	Medial region tibia		
			Medial region tibia			
	LTF	Lateral weight-bearing femur	Lateral weight-bearing femur	lateral weight-bearing Femur	-	-
		Lateral region tibia	Lateral posterior region femur	Lateral region tibia		
			Lateral region tibia			
	PF	Medial region patella	Superior region patella	Medial region patella	-	-
		Lateral region patella	Inferior region patella	Lateral region patella		
		Medial trochlea femur	Medial region patella	Medial trochlea femur		
		Lateral trochlea femur	Lateral region patella	Lateral trochlea femur		
			Medial trochlea femur			
			Lateral trochlea femur			
	Other	-	-	Subspinous region tibia	**-**	Hoffa's fat pad (infrapatellar)
						Medial posterior-condylar region
						Lateral posterior-condylar region
						Medial recess
						Lateral recess
Score (per region)		0 = none	0 = none	0 = none	0 = physiological amount^a^	0 = absent
		1 = <10% of surface area	1 = mild	1 = <10% of bone volume	1 = small^b^	1 = present
		2 = 10 to 75% of surface area	2 = moderate	2 = 10 to 25% of bone volume	2 = medium^c^	
		3 = >75% of surface area	3 = severe	3 = >25% of bone volume	3 = large^d^	
Details			An osteophyte must be visible on two consecutive slices. Largest osteophyte within region scored	If BML span more than one region, then full size of BML is attributed to region that is most involved		

BML, bone marrow lesions; LTF, lateral tibiofemoral compartment; MTF, medial tibiofemoral compartment; PF, patellafemoral compartment. ^a^In supra-patellar bursa only. ^b^Fluid continuous in retropatellar space. ^c^With slight convexity of suprapatellar bursa. ^d^Evidence of capsular distention.

### Statistical analysis

Firstly, descriptive statistics were calculated. Secondly, linear and logistic regression analyses were performed for associations of tissue abnormalities (independent variables) with biomechanical factors and physical examination findings (dependent variables), for continuous and dichotomous scales respectively. Compartment-specific scores for JSW and osteophytes, detected by CR, were dichotomized by combining scores 0 and 1 (that is, only minute abnormality) and combining scores 2 and 3 (that is, at least definite abnormality) for each compartment separately. Region-specific scores (0 to 3) for cartilage integrity, osteophytes and BML, detected by MRI, were summed into compartment-specific scores [27,43-45]. Knee-specific scores for MRI-based effusion were dichotomized, by combining scores 0 and 1 (physiological amount/small effusion) and by combining scores 2 and 3 (medium/large effusion) [28,46]. Region-specific scores for MRI-based synovitis were also dichotomized into one knee-specific score (no synovitis at all vs. synovitis present in at least one region). Regression analyses were performed univariably as well as multivariably with adjustment for age, gender and duration of knee symptoms. Standardized regression β coefficients and *P *values were estimated for linear regression analyses; odds ratios and *P *values were estimated for logistic regression analyses. Statistical significance was accepted at *P *<0.05. All analyses were performed using PASW Statistics 18.0 (SPSS Inc., Chicago, IL, USA).

## Results

From a total of 112 potential candidates that participated in a randomized controlled trial [34] from January 2010, seven persons were excluded (because MRI could not be scheduled before the start of the trial). Patient characteristics of the study sample (*n *= 105) are presented in Table 2. In general, study participants demonstrated multiple severe tissue abnormalities, detected by both CR and MRI, indicating an advanced OA group. An overview of all study findings is presented in Table 3.

**Table 2:** Descriptive data of study population (*n *= 105)

	Mean ± SD	*n *(%)
**Demographics**		
Age (years)	61.4 ± 6.9	
Gender (female)		73 (70)
Duration of knee complaints (years)	11.3 ± 9.3	
Body mass index (kg/m^2^)	29.1 ± 4.7	
Radiographic severity^a^		
K-L score 0/1		32 (31)
K-L score 2		28 (27)
K-L score 3		26 (25)
K-L score 4		19 (18)
Pain severity (NRS, 0 to 10)	5.1 ± 2.1	
**Biomechanical factors**		
Quadriceps muscle strength (Nm/kg)^a^	0.89 ± 0.47	
Hamstrings muscle strength (Nm/kg)^a^	0.61 ± 0.26	
Proprioceptive accuracy (degrees)^a^	2.9 ± 1.9	
Varus-valgus laxity (degrees)^a^	6.9 ± 2.8	
**Physical examination findings**		
Bony tenderness (yes)^a^		75 (71)
Crepitus (yes)^a^		86 (82)
Bony enlargement (yes)^a^		12 (11)
Palpable warmth (yes)^a^		4 (4)
**Tissue abnormalities, detected by CR**	**Median (range)**	***n***
Joint space width^a^		
MTF (0, 1, 2, 3)^b^		5, 29, 31, 40
LTF (0, 1, 2, 3)^b^		68, 20, 10, 7
PF (0, 1, 2, 3)^b^		31, 44, 22, 8
Osteophytes^a^		
MTF (0, 1, 2, 3)^c^		21, 57, 24, 3
LTF (0, 1, 2, 3)^c^		43, 49, 11, 2
PF (0, 1, 2, 3)^c^		11, 67, 25, 2
**Tissue abnormalities, detected by MRI**		
Cartilage integrity^a^		
MTF (0 to 6)	4 (0 to 6)	
LTF (0 to 6)	2 (0 to 6)	
PF (0 to 12)	2 (0 to 12)	
Osteophytes^a^		
MTF (0 to 9)	3 (0 to 9)	
LTF (0 to 9)	2 (0 to 9)	
PF (0 to 18)	5 (0 to 12)	
Bone marrow lesions^a^		
MTF (0 to 6)	1 (0 to 6)	
LTF (0 to 6)	0 (0 to 6)	
PF (0 to 12)	0 (0 to 8)	
Effusion (0, 1, 2, 3)^a,d^		34, 31, 27, 13
Synovitis^a ^presence, *n *(%)		
In at least one region		36 (34)
In Hoffa's fat pad		31 (30)
In medial posterior-condylar region		13 (12)
In lateral posterior-condylar region		12 (11)
In medial recess		6 (6)
In lateral recess		7 (7)

CR, conventional radiography; K-L, Kellgren-Lawrence; LTF, lateral tibiofemoral compartment; MRI, magnetic resonance imaging; MTF, medial tibiofemoral compartment; NRS: numeric rating scale; PF, patellafemoral compartment; SD, standard deviation. ^a^Data from index knee. ^b^0 = none, 1 = minute narrowing (0 to 33%); 2 = definite narrowing (33 to 66%); 3 = ankylosis (66 to 99%). ^c^0 = none, 1 = minute, 2 = definite, 3 = large. ^d^0 = none, 1 = mild; 2 = moderate; 3 = severe.

**Table 3:** Summary of study findings (significant associations*)

	Associations with MRI features	Associations with radiographic features
Biomechanical factors		
Quadriceps strength	Cartilage integrity in PF compartment, effusion and synovitis associated with lower quadriceps strength	Joint space width in PF compartment associated with lower quadriceps strength
Hamstrings strength	-	-
Proprioceptive accuracy	-	-
Laxity	Cartilage integrity in MTF compartment associated with lower varus-valgus laxity	Joint space width in MTF compartment associated with lower varus-valgus laxity; joint space width in LTF compartment and osteophytes in LTF compartment associated with higher varus-valgus laxity
Physical examination findings		
Bony tenderness	-	-
Crepitus	Cartilage integrity in LTF compartment, osteophytes in MTF, LTF and PF compartment and effusion associated with presence of crepitus	-
		
Bony enlargement	-	-
Palpable warmth	-	-

LTF, lateral tibiofemoral compartment; MRI, magnetic resonance imaging; MTF, medial tibiofemoral compartment; PF, patellafemoral compartment. *P <0.05.

**Figure 1:**
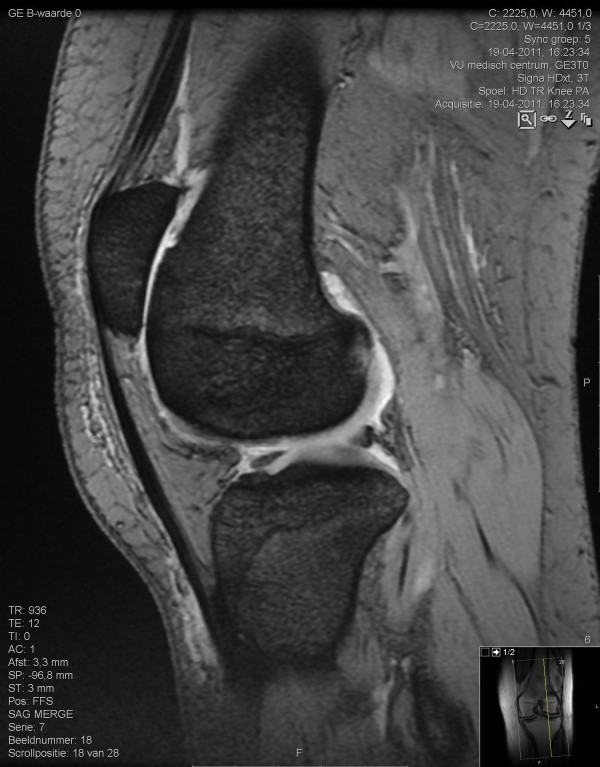
**Reduced cartilage integrity in the patellafemoral compartment**. Sagittal combined multi-echo gradient echo 3.0 Tesla magnetic resonance imaging scan.

**Figure 2:**
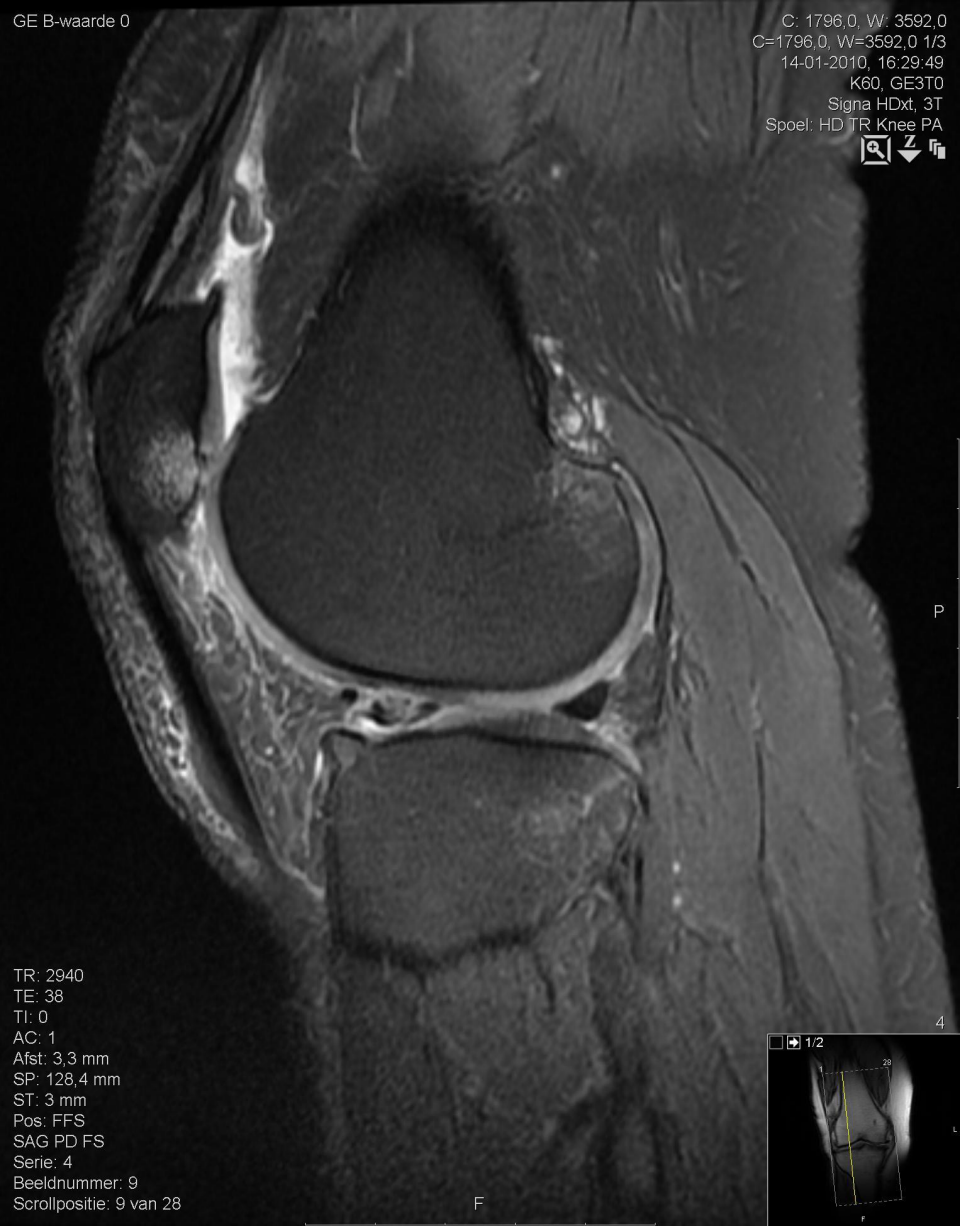
**Infrapatellar synovitis**. Fat-suppressed sagittal proton density-weighted turbo spin-echo 3.0 Tesla magnetic resonance imaging scan.

One feature from CR (JSW in the PF compartment) was found to be significantly associated with lower quadriceps strength (β = -0.18, *P *= 0.030), as shown in Table 4. Three CR features were related to varus-valgus laxity; namely, JSW (β = 0.26, *P *= 0.004) and osteophytes (β = 0.26, *P *= 0.005) in the LTF compartment related to higher laxity, and JSW in the MTF compartment related to lower laxity (β = -0.22, *P *= 0.016). Three MRI features were significantly associated with lower quadriceps strength - namely, cartilage integrity (that is, reduced cartilage thickness) in the PF compartment (β = -0.17, *P *= 0.041) (see Figure 1), effusion (β = -0.16, *P *= 0.049) and synovitis (β = -0.21, *P *= 0.011) (see Figure 2) - while a borderline significant association was found for MTF cartilage integrity (β = -0.15, *P *= 0.073). We also found an association between MRI-detected cartilage integrity in the MTF compartment and laxity (β = -0.22, *P *= 0.017).

**Table 4:** Regression analyses^a ^of association between tissue abnormalities detected by CR and/or MRI and biomechanical factors

		Quadriceps strength (Nm/kg)	Hamstrings strength (Nm/kg)	Proprioceptive accuracy (degrees)	Varus-valgus laxity (degrees)
**Conventional radiography**					
JSW^b^	MTF	-0.12 (0.16)	0.02 (0.84)	-0.03 (0.78)	**-0.22 (0.02)**
	LTF	-0.07 (0.37)	-0.03 (0.71)	-0.08 (0.42)	**0.26 (<0.01)**
	PF	**-0.18 (0.03)**	0.09 (0.28)	-0.03 (0.80)	-0.14 (0.13)
Osteophyte^b^	MTF	-0.03 (0.69)	0.04 (0.66)	-0.10 (0.31)	0.07 (0.47)
	LTF	-0.06 (0.51)	0.06 (0.46)	-0.05 (0.63)	**0.26 (<0.01)**
	PF	-0.09 (0.30)	0.06 (0.44)	-0.09 (0.38)	-0.08 (0.37)
**Magnetic resonance imaging**					
Cartilage integrity	MTF	-0.15 (0.07)	0.00 (0.97)	0.11 (0.25)	**-0.22 (0.02)**
	LTF	-0.05 (0.55)	0.09 (0.30)	0.05 (0.61)	0.07 (0.44)
	PF	**-0.17 (0.04)**	0.10 (0.24)	-0.02 (0.86)	-0.11 (0.23)
Osteophytes	MTF	-0.05 (0.56)	0.11 (0.18)	0.08 (0.43)	-0.08 (0.36)
	LTF	-0.08 (0.33)	0.05 (0.58)	0.02 (0.88)	0.02 (0.87)
	PF	-0.12 (0.15)	-0.01 (0.95)	-0.03 (0.77)	-0.17 (0.06)
BML	MTF	0.03 (0.76)	0.12 (0.13)	0.16 (0.11)	-0.06 (0.48)
	LTF	-0.05 (0.54)	0.12 (0.13)	-0.05 (0.62)	0.12 (0.18)
	PF	0.01 (0.91)	0.16 (0.06)	-0.17 (0.08)	-0.12 (0.19)
Effusion^c^		**-0.16 (0.05)**	-0.08 (0.34)	0.11 (0.26)	0.08 (0.40)
Synovitis^d^		**-0.21 (0.01)**	-0.11 (0.17)	0.03 (0.76)	-0.02 (0.83)

Data presented as standardized regression coefficient β (*P *value). Significant associations (*P *<0.05) in bold. BML, bone marrow lesion; CR, conventional radiography; JSW, joint space width; LTF, lateral tibiofemoral compartment; MRI, magnetic resonance imaging; MTF, medial tibiofemoral compartment; PF: patellafemoral compartment. ^a^Adjusted for age, gender and duration of knee symptoms. ^b^Definite/severe abnormality compared with none/mild abnormality (reference group). ^c^Medium/large effusion compared with no/small effusion (reference group). ^d^Presence of synovitis in at least one region compared with absence of synovitis (reference group).

No associations were found between features from CR and findings from physical examination, as shown in Table 5. On the contrary, multiple MRI features (that is, LTF cartilage integrity, osteophytes in MTF, LTF and PF compartments and effusion) were found to be significantly associated with crepitus, while the association with MTF cartilage integrity was of borderline significance (*P *= 0.050).

**Table 5:** Regression analyses^a ^of association between tissue abnormalities from CR and/or MRI and physical examination findings

		Bony tenderness (presence)	Crepitus (presence)	Bony enlargement (presence)	Palpable warmth (presence)
**Conventional radiography**					
JSW^b^	MTF	0.90 (0.82)	2.13 (0.15)	2.22 (0.34)	0.44 (0.43)
	LTF	0.96 (0.95)	4.24 (0.18)	1.70 (0.50)	n/a
	PF	1.80 (00.27)	1.15 (0.82)	3.09 (0.09)	2.72 (0.35)
Osteophytes^b^	MTF	0.77 (0.59)	1.40 (0.58)	0.78 (0.74)	0.90 (0.93)
	LTF	0.85 (0.81)	n/a	1.29 (0.80)	2.33 (0.53)
	PF	1.34 (0.58)	1.45 (0.56)	1.89 (0.34)	3.52 (0.23)
**Magnetic resonance imaging**					
Cartilage integrity	MTF	0.89 (0.25)	1.25 (0.05)	1.18 (0.26)	0.92 (0.72)
	LTF	0.88 (0.27)	**1.61 (0.02)**	1.26 (0.15)	0.75 (0.38)
	PF	1.03 (0.74)	1.07 (0.49)	0.94 (0.60)	1.30 (0.11)
Osteophytes	MTF	0.92 (0.32)	**1.34 (0.02)**	1.05 (0.69)	1.24 (0.31)
	LTF	1.06 (0.60)	**1.57 (0.01)**	1.29 (0.08)	1.19 (0.48)
	PF	1.09 (0.20)	**1.20 (0.04)**	0.99 (0.90)	1.30 (0.17)
BML	MTF	0.97 (0.76)	1.16 (0.27)	1.01 (0.94)	1.04 (0.87)
	LTF	0.91 (0.53)	1.38 (0.26)	1.45 (0.05)	1.03 (0.95)
	PF	0.84 (0.21)	1.73 (0.12)	0.66 (0.28)	1.29 (0.28)
Effusion^c^		0.75 (0.52)	**7.05 (0.01)**	1.10 (0.89)	5.93 (0.14)
Synovitis^d^		0.84 (0.70)	2.14 (0.21)	1.30 (0.70)	n/a

Data presented as odds ratio (*P *value). Significant associations (*P *<0.05) in bold. BML, bone marrow lesion; CR, conventional radiography; JSW, joint space width; LTF, lateral tibiofemoral compartment; MRI, magnetic resonance imaging; MTF, medial tibiofemoral compartment; n/a, not applicable because of empty cells in analysis; PF: patellafemoral compartment. ^a^Adjusted for age, gender and duration of knee symptoms. ^b^Definite/severe abnormality compared with none/mild abnormality (reference group). ^c^Medium/large effusion compared with no/small effusion (reference group). ^d^Presence of synovitis in at least one region compared with absence of synovitis (reference group).

## Discussion

This is the first knee OA study exploring associations between multiple tissue abnormalities, biomechanical factors and physical examination findings. The study provided several clinically relevant findings. Firstly, the clinically relevant and new finding that high-resolution 3.0 Tesla MRI-detected effusion and synovitis, associated with quadriceps weakness. Secondly, several tissue abnormalities (that is, cartilage integrity, osteophytes and effusion), but only when detected by MRI, were found to be associated with the presence of crepitus. Thirdly, we found associations of cartilage integrity with quadriceps weakness and reduced varus-valgus laxity.

The present explorative study showed only a limited amount of significant associations, which indicates discordance between tissue abnormalities and clinical features in knee OA patients. The lack of significant associations between radiographic and clinical features is not surprising, as the discordance between radiographic and clinical OA [13] is well known and, at least partly, related to the heterogeneity of OA. However, since pain severity has been found to be more closely linked to MRI features than to CR features [47], we were surprised by the limited amount of significant associations between MRI features, biomechanical factors and physical examination findings.

High-resolution MRI and CR provided similar patterns of association. Firstly, reduced PF cartilage integrity, both MRI based and CR based, was associated with quadriceps weakness, which confirms previous studies [18,20,21]. Secondly, MRI-based and CR-based MTF cartilage integrity loss was related to lower varus-valgus laxity. Although these associations were weak and inconsistent with previous studies [8,22,23], they might be indicative for an ankylosing effect of end-stage cartilage integrity (that is, reduced joint motion due to bone-to-bone) [48]. Others suggested that cartilage loss results in higher laxity due to reduced tension on ligaments (pseudo-laxity [8]), which might underlie our finding of reduced LTF cartilage integrity (but only on CR) associated with higher laxity. Future studies are needed to clarify the association between cartilage integrity and laxity. Thirdly, neither features from MRI nor from CR were significantly related to hamstrings strength and proprioceptive accuracy. Finally, no associations were found between tissue abnormalities (MRI and CR) and physical examination findings (except for crepitus), which might, at least partly, be explained by the low proportion of persons with bony enlargement (11%) or palpable warmth (4%) in our cohort. These similar patterns of findings from CR and MRI were determined in a study sample of patients with advanced knee OA with knee complaints for more than 10 years on average. Since MRI is able to detect tissue abnormalities at a much earlier stage of the disease than CR [31], a different pattern of associations with clinical features may possibly be found in an early OA sample. On the other hand, two results from our study may be indicative for an additional value of MRI over CR. Firstly, MRI-based effusion and synovitis, which cannot be detected by CR, were found to be significantly associated with quadriceps weakness. Secondly, crepitus of the knee was associated with multiple MRI features (that is, LTF cartilage integrity, osteophytes in all three compartments and effusion), similar to a recently conducted population-based study [33], but was not associated with any feature from CR. This indicates that MRI seems to be able to visualize features underlying crepitus, while CR is not.

A new and potentially important finding from our study is the association of OA-related inflammation (effusion and/or synovitis) with quadriceps weakness, which is in line with previous experimental studies demonstrating an effect of effusion on quadriceps function [49-51]. Quadriceps muscles are considered the most important muscles for knee movements, stabilization and shock absorption [11]. Persons with synovitis and/or effusion had significantly lower quadriceps strength compared with persons without synovitis/effusion. In secondary analyses, similar results were yielded after adjustment for pain severity, indicating that pain does not explain the association between inflammation and quadriceps weakness. Because inflammation of the synovial membrane had mostly been identified in the infrapatellar region (that is, in 86% of persons with synovitis), which is adjacent to the patellar tendon of the quadriceps muscles, it seems plausible that quadriceps function is affected by inflammatory processes nearby the patella. In addition, inflammation may occur inside the muscle as well, which could result in decreased muscle strength [52]. Effusion is presumed to cause muscle weakness by muscle reflex inhibition due to increased intra-articular pressure [49-51]. Although knee joint inflammation has been suggested to also affect proprioceptive accuracy [7], we were not able to demonstrate this. A possible explanation could be that our study participants demonstrated relatively healthy proprioceptive accuracy. Previous studies provided conflicting results for the role of non-inflammatory effusion (that is, saline injections) in proprioceptive accuracy [53,54]. Future studies need to focus on OA-related inflammation, instead of non-inflammatory injections, to clarify the role of inflammation in proprioception.

The associations between effusion, synovitis and quadriceps weakness could be highly relevant for selecting knee OA treatment. If inflammatory processes underlie quadriceps weakness, regular quadriceps strengthening exercises are not likely to be beneficial and anti-inflammatory therapy might be needed first. This implication is in line with a recent study in which patients treated with both NSAIDs and exercises improved more in muscle strength compared with patients treated with exercises only [55]. In addition, our data revealed that physical examination of the knee strongly underestimated the prevalence of inflammation of the knee, since warmth was palpated in only 4% of our participants, compared with a prevalence of 34% for synovitis and 39% for medium/large effusion, detected by MRI. This implies that MRI may have additional value for clinical assessment in patients with inflammation. Our findings also emphasize that OA is not only characterized by cartilage degeneration and bony changes but also by inflammatory changes, which may point out the importance of anti-inflammatory therapies in knee OA.

Our study design has some limitations that need to be noticed. Firstly, we did not use contrast-enhanced MR imaging techniques to minimize risks for participants (for example, risk of allergic reactions, nephropathy). Because of the well-known superiority of contrast-enhanced MRI for synovitis detection [56], it is remarkable that even without contrast infusions we were able to detect an association of both effusion and synovitis with quadriceps weakness. Although noncontrast-enhanced MRI demonstrated lower specificity for detecting synovitis compared with contrast-enhanced MRI, meaning that signal intensity alterations do not always represent synovitis, it is also been found to be a highly sensitive technique (≈100% sensitivity) for synovitis detection [57]. This implies that the prevalence of synovitis in our study could be an overestimation, but that all persons with actual synovitis have presumably been detected. Secondly, we are not sure whether the power of our study was sufficient. Most MRI studies included large cohorts (*n *>200), while our study consists of 105 participants. This sample size may have resulted in loss of statistical power. In addition, participants had been selected based on the presence of knee joint instability, since they participated in a study on the effectiveness of a knee stabilization exercise program, which may have introduced selection bias in the present study. Thirdly, our study design was cross-sectional with no control group, while a longitudinal design, preferably using a control group of patients at risk, is necessary to unravel interactions between tissue abnormalities, biomechanical factors and physical examination findings. However, this study has a unique design because it is the first study we are aware of in which associations could be explored between both radiography and MRI with biomechanical and physical examination features in a knee OA cohort.

## Conclusions

This explorative study detected several new and clinically relevant findings, including associations of MRI-based effusion/synovitis with quadriceps weakness. Inflammation was detected in over one-third of the participants, emphasizing the inflammatory component of OA and a possible important role of anti-inflammatory therapies in knee OA. In general, OA-related tissue abnormalities of the knee, even those detected by MRI, were found to be discordant with biomechanical and physical examination features. As this is an explorative study, replication in future research is needed.

## Abbreviations

BML: bone marrow lesions; CR: conventional radiography; JSW: joint space width; LTF: lateral tibiofemoral; MRI: magnetic resonance imaging; MTF: medial tibiofemoral; NSAID: nonsteroidal anti-inflammatory drug; OA: osteoarthritis; PF: patellafemoral.

## Competing interests

The authors declare that they have no competing interests.

## Authors' contributions

JK, JD and WFL were responsible for conception and design. JK, JD, JPK, MvdL, MvdE, DR, REV, MG, LDR, MPMS and WFL were responsible for acquisition of data or analysis and interpretation of data. All authors were responsible for drafting the article or revising it critically for important intellectual content. All authors read and approved the manuscript for publication. JK takes full responsibility for the integrity of the work as a whole, from inception to finished article.
